# Supplementary material for: Evaluation of Peregrinus maidis transformer-2 as a target for CRISPR-based control
Source: PLoS One. 2024 Apr 18;19(4):e0295335. doi: 10.1371/journal.pone.0295335 (PMC11025951; doi:10.1371/journal.pone.0295335)
Supplement: S1 Table — (DOCX) [file pone.0295335.s001.docx]

**S1 Table. Primers and gRNAs used in this study.**

^a^ The sequence of the T7 “tail” (primer site for RNA synthesis) is underlined.

| **Application** | **Gene** | **Sequence (5’ → 3’)** | **Product length** |
| --- | --- | --- | --- |
| **Primers**  1^st^-round PCR  (template for  nested PCR) | *Pmtra-2* | F: TCTCGGTTGTGTAGATGTTCA  R: TTGTCTCACAGCAGAGCATA | 420 bp |
|  | *EGFP* | F: ATGGTGAGCAAGGGCGAGGAGC  R: TTTACTTGTACAGCTCGTCCATGC | 721 bp |
| **T7-tailed^a^ Primers**  2^nd^-round PCR  (template for  dsRNA synthesis) | *Pmtra-2* | T7F: TAATACGACTCACTATAGGGTGTTTCCAAGATGGCGTC  T7R: TAATACGACTCACTATAGGGCAGCAGAGCATAACAGAGAA | 389 bp |
|  | *EGFP* | T7F: TAATACGACTCACTATAGGGCACAAGTTCAGCGTGTCCG  T7R: TAATACGACTCACTATAGGGTGCCGTTCTTCTGCTTGTC | 449 bp |
| **Primers**  qRT-PCR | *Pmtra-2* | F: ACGATATCCTCCTCCCCT  R: GACGTATCAGGGTGGACT | 158 bp |
|  | *RPL10* | F: CGCCAACAAGTACATGGT  R: TCCAAAGGCACCTCTCAT | 148 bp |
| **Guide RNAs** | *Pmtra-2* | gRNA1: AGAGCACACACGCCAACUCC  gRNA2: GAUUGACGGACGACGUAUCA | N.A. |
| **Primers**  knockout confirmation | *Pmtra-2* | F: AGGACAACCCTCTACCAG  R: TTGCCTGACAGTTCTTGG | 492 bp |
