# Supplementary material for: Evaluation of Peregrinus maidis transformer-2 as a target for CRISPR-based control
Source: PLoS One. 2024 Apr 18;19(4):e0295335. doi: 10.1371/journal.pone.0295335 (PMC11025951; doi:10.1371/journal.pone.0295335)
Supplement: S1 File — (DOCX) [file pone.0295335.s002.docx]

**S1 File. *Pmtra-2* sequences.**

The expected deletion between the cut sites of two gRNAs is in red.

>Pmtra-2_167888_2

CCATGTTTTAGAAAACAGAACACACGGAACCATTTTCTTGTTTGTTGTATTTGTGTATTTTCTAATTTGGCGTCATCGGTGTTGCTCATAAACTTAGATTGCACTCAAATTTTTAGTAAGGATATCTTCAAATATGAGTGATAGAGAAGATCACGATCTTATTGCTGAGCACAGTCGAGAGGGCAGCGTTGTCTCACAGCAGAGCATAACAGAGAAAAAAGTCATTTATAGGTCGAGATCCAGTGAGAAGGGCAGCGATCGTGGCAGGTCAAGGTCACGTTCGGGAAGCGCCGGTGGTGGGGGAGATCACAAGAGCCGCAGTAGACGCGAGTATTCGGGCTCCAGAAGCAGAAGTAGATCGCGCAGTCGTAGATCGCGTCGCAACAGCAGTCGCTATCGCTCGAGGTCGCGTTCGTCGCGTCGCTACAAGGCACGCTACTCGTACAGTCGCTCGCGATCTGGATCGTCGCGCGATGGAGAGGGCGATGGCTTCCATTCGCACTCGCGGAGTCCGATGTCGACCAGGCGACGCCATCTTGGAAACAGGGACAACCCTCTACCAGGCAAGTGTCTGGGTGTGTTTGGTTTGAACATCTACACAACCGAGAACCAATTGTTGGACATTTTCTCGAAGTACGGCCCCGTTGACAAGGTGCAGGTGATTATTGACGCCAAATCGGGCCGGTCGCGTGGCTTTTGCTTTGTCTATTTCGAGAATCCGGAAGACGCCAAAGTGGCGAAAGACCAGTGCTCGGGAATGGAGATTGACGGACGACGTATCAGGGTGGACTATTCGATAACGACAAGAGCACACACGCCAACTCCCGGCATCTACATGGGAAAACCCACCTACATGGAAGAACGCGGATGGAGGGGCAATAGGGATTATAATGACGATTACTATGGTGGAAGTAGGGGAGGAGGATATCGTACCAGTTACAGGAGCAGTTACCGACGATCACCGTCCCCCTACTATAGGCGGGGCAATCGCTACATGAGATCAAGGTCGCGCTCATACTCGCCACGTCGTTACTAAACTGTGATGGGTTTTCATGATTGACGAATCAGATGTCAGACATTGGCTTCCTAACGTCGAGATCCTAACAGCTGACTGGAAAGAAGGCTGACTCCCGTTCAGC

>Pmtra-2_167888_1

ACAGAACACACGGAACCATTTTCTTGTTTGTTGTATTTGTGTATTTTCTAATTTGGCGTCATCGGTGTTGCTCATAAACTTAGATTGCACTCAAATTTTTAGTAAGGATATCTTCAAATATGAGTGATAGAGAAGATCACGATCTTATTGCTGAGCACAGTCGAGAGGGCAGCGTTGTCTCACAGCAGAGCATAACAGAGAAAAAAGTCATTTATAGGTCGAGATCCAGTGAGAAGGGCAGCGATCGTGGCAGGTCAAGGTCACGTTCGGGAAGCGCCGGTGGTGGGGGAGATCACAAGAGCCGCAGTAGACGCGAGTATTCGGGCTCCAGAAGCAGAAGTAGATCGCGCAGTCGTAGATCGCGTCGCAACAGCAGTCGCTATCGCTCGAGGTCGCGTTCGTCGCGTCGCTACAAGGCACGCTACTCGTACAGTCGCTCGCGATCTGGATCGTCGCGCGACGGAGAGGGCGATGGCTTCCATTCGCACTCGCGGAGTCCGATGTCGACCAGGCGACGCCATCTTGGAAACAGGGTTTCCGGCTACCATGGCGAGAGTTATCAAGATAATCAACAGTCAAAAGACAACCCTCTACCAGGCAAGTGTCTGGGTGTGTTTGGTTTGAACATCTACACAACCGAGAACCAATTGTTGGACATTTTCTCGAAGTACGGCCCCGTTGACAAGGTGCAGGTGATTATTGACGCCAAATCGGGCCGGTCGCGTGGCTTTTGCTTTGTCTATTTCGAGAATCCGGAAGACGCCAAAGTGGCGAAAGACCAGTGCTCAGGAATGGAGATTGACGGACGACGTATCAGGGTGGACTATTCGATAACGACAAGAGCACACACGCCAACTCCCGGCATCTACATGGGAAAACCCACCTACATGGAAGAACGCGGATGGAGGGGCAATAGGGATTATAATGACGATTACTATGGTGGAAGTAGGGGAGGAGGATATCGTACCAGTTACAGGAGCAGTTACCGACGATCACCGTCCCCCTACTAGTAGGCGGGACGGTGATCGTCGGTAACTG
