# Supplementary material for: Evaluation of Peregrinus maidis transformer-2 as a target for CRISPR-based control
Source: PLoS One. 2024 Apr 18;19(4):e0295335. doi: 10.1371/journal.pone.0295335 (PMC11025951; doi:10.1371/journal.pone.0295335)
Supplement: S2 File — (DOCX) [file pone.0295335.s003.docx]

**S2 File. Sequencing result from 2^nd^-round PCR product.**

>Pmtra-2_amplicon

AAAAGTCATTTATAGGTCGAGATCCAGTGAGAAGGGCAGCGATCGTGGCAGGTCAAGGTCACGTTCGGGAAGCGCCGGTGGTGGGGGAGATCACAAGAGCCGCAGTAGACGCGAGTATTCGGGCTCCAGAAGCAGAAGTAGATCGCGCAGTCGTAGATCGCGTCGCAACAGCAGTCGCTATCGCTCGAGGTCGCGTTCGTCGCGTCGCTACAAGGCACGCTACTCGTACAGTCGCTCGCGATCTGGATCGTCGCGCGATGGAGAGGGCGATGGCTTCCATTCGCACTCGCGGAGTCCGATGTCGACCAGGCGACGCCATCTTGGAAACA
